# Perturbation of 3D nuclear architecture, epigenomic aging and dysregulation, and cannabinoid synaptopathy reconfigures conceptualization of cannabinoid pathophysiology: part 2—Metabolome, immunome, synaptome

**DOI:** 10.3389/fpsyt.2023.1182536

**Published:** 2023-10-03

**Authors:** Albert Stuart Reece, Gary Kenneth Hulse

**Affiliations:** ^1^Division of Psychiatry, University of Western Australia, Crawley, WA, Australia; ^2^School of Medical and Health Sciences, Edith Cowan University, Joondalup, WA, Australia

**Keywords:** cannabis, cannabinoid, genotoxicity, epigenotoxicity, transgenerational inheritance

## Abstract

The second part of this paper builds upon and expands the epigenomic-aging perspective presented in Part 1 to describe the metabolomic and immunomic bases of the epigenomic-aging changes and then considers in some detail the application of these insights to neurotoxicity, neuronal epigenotoxicity, and synaptopathy. Cannabinoids are well-known to have bidirectional immunomodulatory activities on numerous parts of the immune system. Immune perturbations are well-known to impact the aging process, the epigenome, and intermediate metabolism. Cannabinoids also impact metabolism via many pathways. Metabolism directly impacts immune, genetic, and epigenetic processes. Synaptic activity, synaptic pruning, and, thus, the sculpting of neural circuits are based upon metabolic, immune, and epigenomic networks at the synapse, around the synapse, and in the cell body. Many neuropsychiatric disorders including depression, anxiety, schizophrenia, bipolar affective disorder, and autistic spectrum disorder have been linked with cannabis. Therefore, it is important to consider these features and their complex interrelationships in reaching a comprehensive understanding of cannabinoid dependence. Together these findings indicate that cannabinoid perturbations of the immunome and metabolome are important to consider alongside the well-recognized genomic and epigenomic perturbations and it is important to understand their interdependence and interconnectedness in reaching a comprehensive appreciation of the true nature of cannabinoid pathophysiology. For these reasons, a comprehensive appreciation of cannabinoid pathophysiology necessitates a coordinated multiomics investigation of cannabinoid genome-epigenome-transcriptome-metabolome-immunome, chromatin conformation, and 3D nuclear architecture which therefore form the proper mechanistic underpinning for major new and concerning epidemiological findings relating to cannabis exposure.

## 1. Introduction

Part 1 of this paper introduced the salience of an epigenomic and aging perspective in understanding cannabinoid pathophysiology (1). Part 2 presents the metabolomic and immunomic basis and underpinning of these changes and then considers their particular application to neurons and the synapse. Thus, this part should be read in conjunction with Part 1 and is properly considered as an extension of it and has been presented in a separate format in view of space considerations.

Much recent attention has focused on the spatial organization of the cell nucleus and the manner in which three dimensional topologically associated domains and transcription factories are epigenetically coordinated to precisely bring enhancers into close proximity with promoters to control gene expression. Many recent papers make the 3D architecture of the nucleus including chromatin conformation a major and principal focus of interest. This perspective is necessarily interactive between genome-epigenome-transcriptome. However, it is increasingly apparent that nuclear events are based squarely on events occurring in the cytoplasm, particularly as relates to metabolomic and immunomic activity which are not only permissive of nuclear changes but determinative of them. Moreover, neuronal and synaptic activity is a special case of these integrated and interactive levels of control and are clearly of particular relevance to the broad spectrum of neuropsychiatric pathologies linked with cannabis exposure, which now include several major neuropsychiatric syndromes and autistic spectrum disorder.

These features also mechanistically underpin several recent large epidemiological studies linking cannabis to the incidence of several cancers (2–12) and numerous congenital anomalies in both USA and Europe (13–19) and with aging syndromes of various types (12, 20), which were detailed further in Part 1.

## 2. Metabolome

The metabolome refers to “the qualitative and the quantitative collection of all low-molecular-weight molecules (metabolites) present in the cell that are participants in general metabolic reactions and that are required for the maintenance, growth, and normal function of a cell” (21). The whole subject of metabolomics is too vast to be covered here. For present purposes, we wish to focus on just three aspects of metabolomics: mitochondriology; code breaking as it relates to the genomic code, histone code, tubulin code, and sugar codes; and the lactylome.

### 2.1. Mitochondria

#### 2.1.1. Mitochondrial inhibition

A large and rich literature demonstrates that the numerous inhibitory activities of cannabinoids on mitochondria have been demonstrated in many laboratories and related to essentially all mitochondrial activities (22–42). A long list of cannabinoids is implicated in these activities including Δ8THC, Δ9THC, Δ10THC, cannabidiol, cannabigerol, and cannabichromene as well as full synthetic cannabinoid agonists such as WIN55,212-2 (28).

Functions inhibited by cannabinoids include such activities as mitochondrial bioenergetics, such as the synthesis of the key enzymes of the electron transport chain (ETC) including the F1 ATPase and many key mitochondrial proteins (43), reduction in the flux through the tricarboxylic acid cycle (TCA), inhibition of ETC complexes I, II, IV, and V activity, lowered NAD^+^/NADH ratio, inhibition of calcium uptake, reduction of the mitochondrial threshold potential for the mitochondrial permeability transition which triggers cellular apoptosis, flux through a new described inner mitochondrial membrane chloride channel, increased reactive oxygen species (ROS) production, especially at complex I, replication of mitochondrial DNA, mitochondrial dynamics (fission and fusion), mitophagy, and biogenesis (28, 43). Most of these actions were triggered through the mitochondrial CB1R and its downstream transduction machinery including a decline in the intracellular cAMP-dependent protein kinase A activity (28). Reduction in the NAD+/NADH ratio impacts gene transcription and expression, cell fate, and differentiation states (28).

Mitochondria also generate and detoxify ROS, synthesize phospholipids, steroids, quinones and haem, perform fatty acid breakdown through β-oxidation, and house the urea cycle (44). Mitochondria have over 1,000 different proteins. The mitochondrial genome is 16,569 bases long and codes for 13 mitochondrial proteins, 22 transfer RNAs, and two ribosomal RNAs (44). Mitochondria are considered major intracellular signaling hubs and coordinate synthesis of nucleotides, lipids, and proteins (45).

Cannabinoids have been shown to impede cellular calcium homeostasis via CB1R, NMDAR, TRPV4, GPR55, and calcium selective Voltage Dependent Anion Channels (VDAC1) of the L-, N-, and P/Q types (28). Release of calcium from mitochondria can occur either through the mitochondrial permeability transition pore or via the Na^+^/Ca^2+^ exchanger (NCLX). Calcium homeostasis in turn controls numerous cell functions including neuronal excitability, muscle contraction, exocytosis, synaptic transmission, gene transcription, cell proliferation, and cytoskeletal movements including cell migration (28). Such changes also control stem cell decisions to multiply, stay dormant, or differentiate (28, 46). Indeed, it has been shown that the cellular NAD^+^/NADH ratio is the key determinant of the rate of embryonic development across multiple species (46).

Many cannabinoids including cannabidiol have been shown to derange mitochondrial structure and cause marked swelling (22, 28, 46).

Importantly, the key master transcription factor for immune activation NF-κB was demonstrated to be sensitive to a buildup of ROS (28).

Both endocannabinoids and phytocannabinoids have been shown to impact mitochondrial bioenergetics, fission and fusion, mitophagy, and mitochondrial transport on intracellular microtubules (28).

Impaired bioenergetic respiratory activity is the initiating event in the caspase-dependent. Cell intrinsic, mitochondrial pathway to cellular apoptosis (28). In neuronal terminals, declining respiratory activity impedes their participation in long-term synaptic depression and potentiation activities, which are key synaptic modulations that underlie the ability to form memories and learn (24, 28).

### 2.2. Astrocytes

It should also be underscored that CB1Rs have been identified on astrocytes. This becomes important when it is understood that astrocytes are now known to play an integral and active part in neurotransmission and form, together with endothelial cells, the “quadripartite synapse” (47, 48).

Indeed, the astrocytic CB1R has emerged as a key player in the control of gliotransmission and the metabolic cooperation between astrocytes and neurons. The CB1R has been described as being the key coordinating factor between gliotransmission and metabolic cooperation in the neuronal-astrocytic dyad (48). Astrocytes also express other cannabinoid receptors including TRPV series, GPR55, and GPR18, but their role has not been well-studied at this point (48).

Astrocytes are bound together via gap junctions and signal to each other by calcium waves. These waves have been shown to be critical in the formation of various kinds of memory including short-term, long-term, working, spatial, and aversive memories (47, 48).

NMDAR activation induces an inward calcium current and is key to synaptic plasticity. Astrocytes synthesize and release glycine and D-serine, which are the co-agonists of the NMDAR (48). Astrocytes also release other gliotransmitters including purines and glutamate. Rising extracellular potassium, released by active neurons, mildly depolarizes astrocytes and strongly stimulates the astrocyte Na^+^/K^+^ ATPase pump and the electrogenic sodium-bicarbonate transporter, which together alkalinize the astrocytes and stimulate glycolysis and lactate release (48). This response also occurs with other neuronal signals such as ammonium and nitric oxide.

Astrocytes have a low concentration of CB1R but are functionally very important (48). Moreover, astrocytes carry the synthetic and metabolizing enzymes for both anandamide (AEA) and 2-arachidonoyl glycerol (2AG) (48). This production is sensitive to external signals. particularly calcium released as a result of neuronal activity.

The role of lactate has metamorphosed from being conceptually considered as a metabolic waste product, to an energy substrate, to an important metabolic signal (47). Astrocytes provide lactate to neurons via an astrocyte-to-neuron lactate shuttle in order to supplement their very high needs for energy, especially during active firing states (47). Reduction in either the MCT2 or MCT4 (medium chain acyl transporters 2 and 4), which are the main lactate channels in neurons and astrocytes, respectively, impedes memory formation and learning. Loss of neuronal MCT2 causes reduced hippocampal neurogenesis and also reduced maturation of newborn hippocampal neuroblasts (47). Astrocyte glycogen metabolism is also important for neuronal memory formation and this is thought to be acting via lactate signaling. Lactate signals are also transduced by the Hydroxycarboxylic acid 1 (HCA1) receptor (48). In this manner, lactate has been shown to be critical to memory formation in many respects (47).

For these reasons, astrocytes are also involved in long-term synaptic depression and potentiation and thus learning and memory. Cannabinoids disrupt these processes in part by inhibiting mitochondrial processes. Inhibition of astrocytic ETC complex I via mitochondrial membrane CB1Rs slows glycolysis, diminishes ROS production, inhibits HIF1α, and thereby reduces synthesis of glycolytic enzymes and glycolytic flux. This, in turn, reduces lactate production and induces bioenergetic and oxidative stress which then impacts memory, social interactions, and behaviors (48).

### 2.3. Mitonuclear communication, mitohormesis, and systemic mitokines

Many studies indicate that mild stresses can be good for organisms (44). Calorie restriction and intermittent fasting are thought to work as effective lifespan extension interventions in this manner in aging research (44). This is known as “hormesis”. When it is induced in mitochondria it is known as mitohormesis (44). Mitochondria are major signaling hubs both to the cell nucleus and to other intracellular organelles such as endoplasmic reticulum.

Some of these stress signals are released systemically, where they are known as mitokines. Such mediators that have been identified in mammals include FGF21, humanin, and GDF15 (44). FGF21 signals to the liver, adipose tissue, and brain (44). When damaged components of mitochondria are released into circulation, they become identified by the body as DAMPs (Damage-Associated Molecular Patterns), which stimulate innate immune pattern recognition receptors. These can include components of the ETC, TCA metabolites, mitochondrial DNA, and the mitochondrial transcription machinery proteins (44). Interestingly, all three of these defined mitokines (FGF21, GDF15, and humanin) increase with age (44). They all improve various measures of health. Thus, the suppression of normal mitochondrial function by numerous cannabinoids can be expected to reverse these effects.

For many reasons, the mitochondria and nucleus need to coordinate their activities. Since most mitochondrial proteins are encoded on nuclear DNA and since many nuclear reactions are dependent on mitochondrially derived energy or substrates of intermediate metabolism, there clearly needs to be a close cooperation and coordination of the activities of these organelles. Therefore, much communication occurs in the anterograde direction from nucleus to mitochondria.

However, communication can also occur in the opposite direction and mitochondria can communicate with the nucleus via malate/aspartate exchange, glyceraldehyde-3-phosphate shuttle, nicotinamide mononucleotide transfer via ROS, by SIRT1, AMPK, and ATFS1, and by substrate and energy supply (45, 49). Key metabolites which signal to the nucleus are acetyl-coenzyme A (acCoA), α-ketoglutarate (αKG), nicotinamide adenine dinucleotide (NAD^+^), succinate, fumarate, and methionine (45). αKG is a required substrate for the major epigenetic demethylation reactions both of DNA—through ten-eleven translocation methylcytosine dioxygenase—and of histones by the Jumonji C(Jmjc) domain containing lysine demethylases (JMJDs) (45). The activity of both TETs and JMJDs is regulated by the ratio of substrates including αKG to inhibitors including succinate, fumarate, and 2-hydroxyglutarate (αHG). In flies, αKG supplementation leads to life span extension by activating AMPK and inhibiting the master anabolic regulator mTOR (45).

NAD^+^ is a key co-substrate in the core reactions of the TCA, ETC, glycolysis, and β-oxidation of fatty acids (50). It is also a core co-reactant in three groups of key epigenetic enzymes, the sirtuins and HDACs, the PARPs (poly-APR-ribose polymerases), and cADP-ribose synthase (CD38), which control gene expression. In yeast, flies, worms, and mice NAD^+^ and sirtuins control aging. Pharmacological supplementation of NAD^+^ in worms extends lifespan via the mitochondria unfolded protein response (UPR^mt^), which is mediated via ATFS1 (45). PARP is also involved in DNA repair, inflammation, and cell death (45).

Methionine is the chief amino acid source of one carbon group for epigenetic methylation reactions. The cytoplasmic folate cycle is coordinated with the methionine cycle to maintain levels of s-adenosyl methionine, which is the primary one carbon donor for these reactions. It is particularly involved in the generation of H3K4me3, which marks transcriptional start sites and is particularly important during embryogenesis. Thus, anything which slows cytosolic one carbon metabolism will necessarily severely impact epigenetic regulation (45).

### 2.4. Code breaking—Genome, histone, tubulin, and sugar codes

#### 2.4.1. Genomic code

It was shown several decades ago that cannabinoids reduce the synthesis of DNA and RNA generally, which necessarily disrupts these key codes (51–53). Moreover, Δ9THC, cannabidiol, and cannabichromene all cause single and double stranded DNA breaks (53–59). Cannabis has long been linked with chromosomal breaks including ring and chain chromosome formation, microchromosomes, and micronuclei (53, 60–63). Broken chromosomes can cause breakage-fusion-bridge cycles that are self-perpetuating (64). Similarly, micronuclei cause chromosomal shattering (“chromothripsis”) which rapidly causes widespread genomic chaos from random repair of the chromosomal fragments to random insertion into other chromosomes (65–70). Cannabis is directly mutagenic and is known to oxidize guanine to oxoguanine, which is a highly mutagenic and oncogenic mutation (54). This has been defined both *in vitro* (54) and in human cannabis users (71). Indeed, in one clinical study, the percentage of damaged and broken DNA included in the comet tail in a comet assay rose from 4.8% in controls to 66.4% in cannabis smokers (71). Moreover, as shown by the Harvard group, DNA breakages cause re-arrangement of the histone machinery on the genome and relocation of major histone complexes (72). This removes histone suppression from silenced parts of the genome, especially repetitive mobile elements and retrotransposons (73–80). This makes the “jumping genes jump” which is a severe cause of genomic instability, neurodevelopmental defects, cancer, and aging (73, 74, 76, 78, 81–83). Since cannabis impedes mitochondrial and metabolic function generally, and these are the substrates upon which genomic maintenance is built, it follows that metabolic-genomic factors also disrupt the genome. For these reasons it appears that cannabis disrupts the genome in many respects.

#### 2.4.2. Histone code

From the above remarks in relation to the profound epigenomic disturbances that are induced by many cannabinoids, it is clear that cannabinoids disrupt the epigenomic code in many ways. This effect is itself compounded when it is appreciated that THC actually reduces the synthesis of histones and some of their key post-translational modifications including phosphorylation and acetylation (84, 85). Since histones control the overall genomic architecture and conformation in 3D space, which in turn controls gene transcription, it is clear that the implications of this are very far reaching indeed. They include neurodevelopmental defects, congenital anomalies, cancer, and cell death. Indeed, the North Carolina group noted more than 10,000 differentially methylated regions in cannabis dependence and in excess of 10,000 differentially methylated regions in cannabis withdrawal (86). Cannabis also disrupts the micro-RNA profile of neurons and other cells (87).

“To set these findings in context it may be noted that Schrott et al. performed a profoundly useful longitudinal epigenomic DNA methylation study of human sperm from cannabis users compared to controls before and after an 11 week period of confirmed abstinence from cannabis (86). The authors found thousands of differentially methylated genes altered between cannabis users and controls many of which improved after the period of abstinence. However, after the abstinence period a largely different set of differentially methylated genes emerged in the sperm methylome. The genes affected related to essentially all tissues, to key genes of the epigenomic machinery, to synapse, brain and organ development and to cell growth including many cancers.”

Moreover, as cannabis clearly ages people (see above section) and as aging has now been shown to be a combined genomic-epigenomic disease (72), this also demonstrates that cannabis broadly disrupts the genome and epigenome in several respects.

#### 2.4.3. Tubulin code

Cannabis has been shown to reduce the overall synthesis of α-, β-, and γ- tubulin (43). By definition, therefore, it necessarily disrupts the tubulin code. Moreover, it also acts epigenomically to disrupt acetylation of tubulin (86). This greatly reduces the tensile strength of polymerized tubulin in microtubules, which increases their risk of fracture when bent as in mitotic spindles. This is then a cause of chromosomes coming loose from the mitotic spindle at anaphase and a proximate cause of chromosomal missegregation and micronucleus formation which drives genomic instability. Another key posttranslational modification of tubulin is glutaminylation. This is performed by enzymes of the tubulin-tyrosine-like (TTLL) family. Eight members of the TTLL family are epigenomically disrupted by cannabis exposure (86). Thus, cannabis disrupts not only the synthesis of tubulin but also two if its principal posttranslation modifications which govern its intracellular address targeting and final functionality. Similarly, cannabis also disrupts actin synthesis both directly and epigenomically (43, 86, 88). Thus, cannabis disrupts the presence and function of both tubulin and actin, which are the two major components of the cellular cytoskeleton and both of which are intimately involved in genomic (microtubules of the mitotic spindle) and epigenomic (nuclear lamina and gene silencing and heterochromatin formation) stability.

#### 2.4.4. Sugar code

The sugar code has been called the third great alphabet of life after the nucleic acid and protein codes (89–92). The addition of glycan moieties is the commonest posttranslational modification of proteins (89, 92). In the sugar code complex, usually polymerized and branched carbohydrate moieties called glycans are added to DNA, RNA, and protein which greatly changes their function (91–93). Immunoglobulin G (IgG) is one such well-studied molecule (94–102). It is known that in this case glycans can change the IgG activity from immunostimulatory to immunosuppressive. Aging and many diseases, including atherosclerosis, dementia, rheumatoid arthritis, systemic lupus erythematosus, and cancer, are linked with various modifications of this glycan code (94–105). Cannabis has been shown to epigenomically perturb the activity of some of the key enzymes involved with writing this code including O-Linked N-Acetylglucosamine (GlcNAc) Transferase (95 differentially methylated regions, DMRs or “hits” in the Schrott epigenomic database), UDP Glucuronosyltransferase Family 1 Member A1 (9 DMRs), beta-1,4-N-Acetyl-Galactosaminyltransferase 2 (2 DMRs), fucosyltransferase (9 DMRs), various mannosyltransferases (Programmed Cell Death 6, Dpy-19 Like C-Mannosyltransferase 3, Transmembrane O-Mannosyltransferase Targeting Cadherins 1; 16 DMRs in all), and various glucuronosyltransferases (LARGE Xylosyl- And Glucuronyltransferase 1, UDP Glucuronosyltransferase Family 1 Member A1, 13 DMRs in all) (86). Thus, it is seen that the sugar code is also disrupted by cannabis.

### 2.5. Lactylation and the lactylome

Lactate is relevant to cannabis medicine because cellular lactate usually rises when oxidative metabolism is inhibited, as many cannabinoids are known to do. Moreover, high levels of lactate have been documented following human cannabis use (106) and, since extracellular and intracellular levels of lactate are closely correlated (107), it follows that cannabis induces a systemically high lactate environment.

It was shown that at about 20 sites on histone H3 and H4 lactylation can occur as a posttranslational modification (108). It was shown in bone marrow-derived macrophages that during the activation phase of the M1 macrophage response to an infection they switched to anaerobic glycolysis and the use of lactate as a primary metabolic fuel (108). Rising levels of lactate caused the “writing” of the lactylation code on H3 and H4 ε-carbon of the lysine tails, which 16–20 h after activation caused an epigenetically induced switch to an M2 anti-inflammatory type and inflammasome inhibition in order to assist resolving the acute infection (108). This temporal delay is referred to as a “lactate clock” (108). It was subsequently shown that the switch in macrophage metabolism was controlled by B-cell adapter for PI3K (BCAP), which is a signal adapter for toll-like receptors (107) and assists with the formation of H3K9ac and H3K18 lactylation.

Lactate lies at the intersection of aerobic and anaerobic metabolism, is a major metabolic fuel, and has been said to be quantitatively more important than glucose (107). Lactate is a recyclable redox buffer and provides metabolic feedback through direct activities, through intra- and extra- cellular acidification, through active and passive lactylation of proteins including histones, and thereby epigenetic regulation of gene transcription which plays key roles in such major cellular events as inflammation, wound healing, ischaemia-reperfusion injury, immune responses, neuronal function including learning and memory, and tumorigenesis (107). Lactate also functions as a signaling molecule and has its own dedicated receptor GPR81 and can traverse cell membrane via the MCT1-4 (monocarboxylate transporters 1–4). The lactylation code can be passively written non-enzymatically or actively written by P300. The lactylation code is erased by HDACs 1–3 and sirtuins 1–3 with the aid of P53. HDAC3 was the most powerful of these erasers. Lactylation takes its places along other posttranslational acyl modifications of histones including formylation, propionylation, butyrylation, crotonylation, 2-hydroxybutyrylation, β-hydroxybutyrylation, succinylation, malonylation, glutarylation, and benzoylation and indeed there is some evidence for cross-talk between these various acylation modifications (107). Thus, lactylation and the many metabolic functions of lactate itself represents a case of metabolic regulation by metabolites (107). Its involvement in epigenomic regulation represents a key intersection of metabolism and gene regulation.

It is well-known that glycolysis can be triggered by hypoxia or irreversible mitochondrial inhibition. However, it has now been shown that it can also be triggered by metabolic reprogramming as induced in cancer cells by the excessive demand for ATP imposed by the highly proliferative state. Lactate rises in other situations of cellular stress such as myocardial infarction, trauma, infection, and heart failure (107).

Whilst the physiological concentration of lactate is 1.5–3.0 millimolar, it can rise as high as 10–40 millimolar within inflammatory sites (107). Lactate is generated from pyruvate at the end of glycolysis irrespective of the local oxygen tension. Pyruvate is transformed into lactate by lactate dehydrogenase (LDH). Pyruvate is irreversibly removed by pyruvate dehydrogenase (PDH) which transforms pyruvate into acetyl-Coenzyme A (acCoA). Thus, the lactate level is determined by the balance between glycolytic flux and PDH activity (107). In cancer cells lactate also arises from glutamine catabolism (107). Lactate also controls mitochondrial oxidative metabolism and redox balance. When mitochondria are stressed, they generate more ROS, which further impairs oxidative metabolism and can be dangerous to the cell. Extracellular lactate can be converted into pyruvate by extracellular lactate oxidaze and catalase (107).

The lactate shuttle operates in tumor cells which shuttles lactate between glycolytic tumor cells and oxidatively metabolizing tumor cells with the effect of stimulating overall tumor growth. It also occurs between exercising skeletal muscle and the heart, in a paracrine manner between cardiomyocytes and fibroblasts, between astrocytes and neurons, and between the proximal and distal tubules in the kidney (107). Lactate can restore acCoA levels and enhance fatty acid synthesis in part by increasing the activity of the key metabolic enzyme acCoA carboxylase. Thus, lactate can act in either an autocrine, paracrine, or endocrine manner.

During sepsis, the high circulating lactate levels can lactylate HMGB1, which can then make its way from the nucleus to the cytoplasm via GPR81 and MCTs. From the cytoplasm it can be released via exosomes from where it can damage endothelial cells, increase vascular permeability, and exacerbate sepsis (107). Lactylation also plays several key roles in the pathogenesis of systemic lupus erythematosus (SLE). During red blood cell development, a regulatory switch in the ubiquitin proteosomal system (UPS) occurs but the UPS becomes lactylated in SLE. This lactylation inhibits its UPS activity so that it is not completely able to clear the mitochondria from the developing haemoblast precursors. Macrophages engulf the faulty red cells and consume the mitochondrial DNA, which generate an immune response to DNA via the powerful cGAS-STING system (107).

As noted above, lactate is generally anti-inflammatory following the very earliest phases of an inflammatory stimulus. It is worth considering some of these effects by cell type. In general terms, these effects occur either because of lactate as a metabolic substrate, via lactate signaling, or from lactylation of key proteins (107).

In macrophages, lactate activates the ERK-Stat3 pathway, GPR132, Notch signaling, HIF1α stabilization, and histone lactylation. Together these effects induce M2 Polarization and increases IL-6, VEGF (stimulating angiogenesis), ARG1, and CCL5 (107).

In T-cells, lactate induces an acidic environment which completely inactivates T-cell signaling machinery, activates PD-1 and PD-L1 pathway, inhibits P38, JNK, and lactate efflux and thus poisons the T-cell, thereby stopping T-cell activation and cytokine mobilization. Taken together these effects decrease effector function and T-cell proliferation and increase PD-1 expression and immune evasion (107).

In dendritic cells, lactate induces an acidic environment, reduces CD1a and increases CD14 expression, and activates GPR81 signaling and lactate, which is important via SLC16A. Together these effects decrease differentiation, decrease IL-12 production, IL-6 release, and TNFα release, and increase kynurenine release (107).

In NK cells, lactate induces an acidic environment and inhibits HDACs, NFAT, NKp-46, and mTOR signaling. Taken together these effects decrease cytolytic function and interferon γ release and increase apoptosis (107).

In mast cells, lactate induces an acidic environment, targets MAS-associated G protein coupled receptor, and inhibits early calcium mobilization and degranulation and also late chemokine/cytokine phases of activation (107).

In Treg cells, lactate induces FOXP3-mediated repression of Myc and modulation of LDH and sustains fatty acid synthesis through acetyl-CoA carboxylase. Together these effects increase proliferation, differentiation, TGFβ, and IL-10. In combination with effects on the above effector cells these effects further reinforce the immunosuppressive phenotype (107).

Through tissue acidification the effects of lactate are proinflammatory in most chronic inflammation scenarios (107).

Lactate is powerfully protumourigenic in many pathways: as a substrate for energy metabolism; by activating PD-1,/PD-L1 pathway, and T-cell apoptosis; synergizing with oncogene Myc; activating oncogene HIF1α and VEGF /VEGFR2; via lactylation of histone and non-histone proteins; inhibiting NFAT, NKp-46, and mTOR signaling; and increasing tumor growth and metastasis, tumor angiogenesis, and tumor invasion including basement membrane remodeling, metalloproteinase activation by epitranscriptomic m6A methylation mRNA of MTLL3, altered tumor immunity, and induction of immunosuppressive tumor environment (107).

Thus, lactate and lactylation represent key therapeutic targets for the development of future therapies (107).

### 2.6. Lactylation in hepatocarcinogensis

A detailed dissection of the role of elevated tissue lactate was made in two series of Hepatitis B-related hepatocellular carcinoma (HCC) patients of 52 and 159 cases each from China (109). High lactate is directly and powerfully immunosuppressive to immunocytes (110, 111).

Lactylation was a posttranslational modification made on lysine residues (denoted as Kla). Lactylation itself was found to usually be an active and regulated process. Many proteins were lactylated by P300 and delactylated by HDAC1-3 (109). Indeed, these researchers found 9,275 sites for lactylation on 6,403 proteins. The enzymes most affected were metabolic enzymes (oxidoreductases and dehydrogenases including mitochondrial complex V and F_0_-F_1_ ATPase, the powerhouse that actually produces mitochondrial ATP at the end of the ETC) and also translational proteins, translation factors, ribosomal proteins, signal receptors, ligases, and cytoskeletal proteins, indicating that lactylation affects many cellular processes including signal transduction, gene expression, and cellular morphology, shape, and motility (109).

The investigators found that the prognosis of patients with high levels of lactylation was much worse than those with lower levels by about 20% at 8 years follow-up (109). A reciprocal relationship was observed between downregulation of normal liver processes and upregulation of oncogenic signaling. Tumor aggressiveness was directly related to the degree of oncogenic skewing of the lactylome (109). Glutamine metabolism was increased as a result of the lactylation of the β-catenin pathway and glycolysis metabolism was increased as a result of the lactylation of the glycolytic pathway.

Branched chain amino acid (valine, leucine, isoleucine, and BCAA) metabolism was also boosted in HCC (109). Via ACAT1 BCAA are an alternative source of acetyl-CoA two carbon skeleton supply for the citric acid supply and also a source for glutamine synthesis. In mouse models of HCC, higher levels of BCAA were correlated with lower survival. ACAT1 was identified in the Schrott epigenomic cannabis screen by two DMRs (86).

Adenylate kinase (AK2) was studied in detail. Heavy lactylation of AK2 was found to be an adverse prognostic factor in terms of survival and hepatic vein thrombosis. The P53 was downregulated in these tumors, partly explaining the poorer prognosis. AK2 also impacted the apoptotic pathway by inhibiting caspase 9 (a final executioner caspase in the cascade) and thereby conferring an anti-apoptotic protection on tumor cells (109). Lactylation of AK2 at K28 in the enzyme active site abolished enzymic activity. Tumors manifesting AK2 K28Kla had increased proliferation, DNA replication, and nucleotide excision repair (a low fidelity DNA repair pathway). AK2 K28Kla was found to drive cell proliferation and cell migration in various HCC cancer cell lines (109).

For these reasons, protein lactylation was shown to be a key posttranslational modification that transduced HCC metabolism into tumor cell behavior and subsequent adverse patient outcomes.

## 3. Immunome

The immunome may be defined as the set of peptides, proteins, receptors, signaling systems, genes, and cells that together comprise the innate and adaptive immune systems (112, 113). Immune processes are highly relevant in many ways as the strongly oxidizing environment of immune and inflammatory processes can induce DNA breaks. So important is immune activity that chronic immune stimulation is now described as one of the pillars of aging (114) and the immunome has now had its own biological clock developed with which to measure organismal biological age (115). Interestingly, most of the predictive power of this clock is related to CXCL9 (115). Chief among the immune pathways is the recently described innate cytosolic nucleic acid sensing and signaling pathway cGAS-STING.

### 3.1. cGAS-STING

#### 3.1.1. Basic function

cGAS-STING is a powerful cytosolic sensor of double stranded DNA (dsDNA) which has been recently described and is of broad relevance to cancer, aging, and genomic stability (116). cGAS also senses R-loops (117–121). Once cGAS binds to dsDNA, its endoplasmic reticulum-bound binding partner STING stimulates a type I interferon response (122). Oligomerization of cGAS, formation of long DNA-protein ladders, and phase separation (in a gel) lead to STING oligomerization and an augmented STING response (122–124). cGAS is activated by DNA in a length-dependent manner and is more powerfully stimulated by longer DNA segments (125). Termination of the cGAS-STING signal occurs by clathrin-associated AP-1 (126). The electron microscopic structure of cGAS-STING has been determined (122, 127–129).

#### 3.1.2. Roles in disease

In addition to innate immunity, cGAS-STING also drives autophagy, cell survival, infection, inflammation, cancer, and senescence pathways (123). cGAS can transfer between cells and activate STING nearby in paracrine fashion (123). cGAS-STING drives aging, senescence, anti-tumor immunity, autoimmune disease, and acute and chronic heart failure inflammatory disorders such as pancreatitis, macular degeneration, alcoholic hepatitis, cancer, metastasis, myocardial infarction, sepsis, systemic lupus erythematosus, and Parkinson's disease (116, 130).

cGAS-STING has been shown to be a major driver of organ pathology in COVID-19 infections (131).

#### 3.1.3. Cancer

cGAS-STING surveils cancer cells. Mice and human cells deficient in cGAS-STING tolerate oncogenic Ras signaling. Neither irradiation nor cancer generate the usual inflammatory responses in cGAS-STING deficient cells. cGAS-STING activity correlates with the inflammatory component of many cancers (116).

cGAS-STING surveillance of micronuclei generates an immune response triggered by genomic instability upon breakdown of the micronuclear envelope (132). Micronuclei can be generated from lagging chromosomes.

cGAS-STING drives the IL-6 dependent survival of chromosomally instable cancers through a chromosomal instability/cGAS-STING/Stat3 /RelB /NF-κB/Stat1/ILK-6/IL-6R/JNK /ASK /cell death pathway (133). This mechanism was found commonly in many cancers that express IL-6R including triple negative breast cancer (133).

cGAS-STING also drives cancer metastasis. Chromosomal missegregation forms micronuclei that trigger cGAS-STING and downstream non-canonical NF-κB signaling that triggers metastasis (134).

In pancreatic cancer cGAS-STING-induced regulatory B-cells compromise NK cells anti-tumor immune response. cGAS-STING promotes immune evasion and metastasis through induction of PD-L1 expression (135). B-cells can also suppress tumor immunity through GABA signaling (136).

#### 3.1.4. Aging

During senescence, reduced lamin B synthesis leads to partial breakdown of the nuclear envelope and blebs of nuclear membrane break off carrying chromosomes. These cytoplasmic chromosomes stimulate cGAS-STING (116). Similar nuclear blebs have also been observed from cannabis exposure (53, 55, 60).

Chronic immunostimulation is a prominent hallmark of aging, particularly the elaboration of the well-characterized Senescence Associated Secretory Phenotype (SASP) made up of growth factors and cytokines (114, 137). cGAS-STING recognizes cytosolic DNA fragments in senescence which generates the SASP and drives senescence with autocrine and paracrine effects (138). This was shown experimentally using irradiation and oncogene induction of senescence pathways (138).

cGAS-STING was shown to be essential for senescence induction. When it was inactivated senescence did not occur and mouse fibroblasts became oncogenically transformed more easily (139). cGAS-STING was required for the induction of senescence by irradiation and cytotoxic therapy. DNA damage produces cytoplasmic DNA which is sensed by cGAS. DDR is a key event in senescence induction. Lung cancers with low cGAS-STING expression have a worse prognosis (139).

YAP/TAZ activity in stromal cells controls cGAS-STING activity and this prevents aging (140).

### 3.2. Cannabinoid immune actions

Immune cells produce endocannabinoids (eCB) and also have receptors and metabolic machinery to transduce and extinguish eCB signals. This implies that eCB's signal through autocrine and paracrine routes to immune cells and their neighbors (141).

Toll-like receptors (TLR) are one of the major receptors of innate immunity and respond directly to a wide variety of exogenous DAMPs and PAMPs. Cannabinoids are generally suppressive of TLR signaling albeit there are many exceptions to this (141). Inflammatory signaling can either stimulate or inhibit ECS signaling via CB1R and CB2R signaling or by changing the levels of activity of eCB metabolic enzymes (141).

When THC was administered to homogenized cultures of rat telencephalic brain it was metabolized by mixed cultures of glia along with neurons (142). GABAergic neurons were most sensitive to a single application of 1 μM THC. After repeated applications of 1–2 μM THC markers of GABAergic, cholinergic and astrocytic damage were greatly elevated (142). IL-6 release was also elevated 4-fold after a single application of THC documenting important pro-inflammatory activity for this major cannabinoid (142).

Many other immune actions of cannabinoids are described which have been reviewed (143–156). Both immunosuppressive (143–145, 157, 158) and immunostimulatory (142, 159–161) actions have been described. Cannabidiol appears to be mainly immunosuppressive in its actions (156, 162). CB1R activation is often immunostimulatory (159–161). CB2R activation is frequently immunosuppressive (152, 163–165).

The immunosuppressive activities of cannabinoids are of clinical interest and importance. The immunosuppressive activities of cannabinoids have been shown to be relevant to different human disorders where heightened immune reactivity is problematic and complicate situations where immune compromise may be cause for concern. Experimental studies have explored the application of cannabidiol to skin graft rejection (166), to protect against the dietary and immune dysregulation induced by a high-fat diet (167), to protect against hypothalamic microgliosis and astrogliosis induced by a high-fat diet (168), to protect against experimentally induced oral mucositis induced by chemotherapy (169), to modulate TLR4 co-receptor signaling and thereby improve morphine mediated analgesia (170), and potentially as an adjunctive application in COVID-19-related cytokine storm (171). Contrariwise, cannabinoids have been found to exacerbate experimental sepsis (172) and to suppress anti-tumor immune responses by inhibiting T-cell Jak/Stat signaling (173).

Human studies have explored the use of cannabinoids in multiple sclerosis (156, 174–180). In general terms, cannabinoids have been found to reduce muscle spasticity (176–181). Cannabidiol has been shown to reduce leukocyte recruitment to plaque inflammatory lesions during peak disease activity (182). However, sedation, psychotomimetic, and gastrointestinal symptoms are often problematic side effects of cannabinoids, particularly in patients who are cannabinoid naïve (174, 176–180).

### 3.3. Long-lasting epigenetic effects in immune stem cells

Whilst there are numerous published examples of the epigenomic effects of cannabinoids on immune cells, detailed consideration of a few key examples illustrates the importance of this feature. One of the long-standing mysteries of adaptive immunity has been the way in which long-term memories of past antigenic exposure can be retained despite the fact that most immunocyte effector cells have only short half-lives. This mystery was elucidated recently by the demonstration that the memories are retained epigenetically in the epigenome of the long-lived immune stem cell population (183, 184). Epigenetic immunological memory storage has been demonstrated in macrophages of the bone marrow, tissue resident macrophages and microglia, monocytes, haemopoietic progenitors, natural killer cells, and innate lymphoid cells (183). Moreover, similar nuclear epigenetic storage of past cell experiences including toxic exposures has been demonstrated on cells of the skin, lung, intestine, pancreas, muscle cells, sperm, endothelial cells, nasal epithelia, Schwann cells, and neurons (183). Moreover, these epigenetic memories have been shown to affect the subsequent risk of diseases such as cancer and Alzheimer's disease. Further, encounters with stress (including the nutrient stress of famine) and environmental toxicants can be passed epigenetically from mother to child *in utero* (183). A broad spectrum of epigenetic machinery is implicated in encoding such memories (183).

One implication of the epigenomic recording of inflammatory memories has been demonstrated in haemopoietic stem cells which become exhausted after recording such encounters (185). This is the mechanistic basis of many of the hematological changes observed in aging including anemias, pancytopaenias, Age-Related Clonal Hematopoiesis (ARCH), leukemogenesis, fatty replacement of the bone marrow, and bone marrow hypocellularity. This study was performed over 1 year in mice, which is equivalent to about 25 years in humans. Thus, these past immune encounters were shown to drive hematological aging (185).

Through blood bank studies, ARCH has recently been shown to be an important risk factor for myocardial infarction, cancer, and all-cause mortality (186). Somatic mutations of DNMT3a are very common in ARCH, although ARCH does not arise for several decades after these are acquired. This has suggested to some investigators that an environmental or inflammatory factor may contribute to oncogenic progression. By studying a mouse line of DNMT3a knockout cells, it was shown that either chronic infection, or just an injection of interferon-γ by itself, was sufficient to cause hypomethylation, reduce cellular differentiation, and lower stress-induced apoptosis rates, which together account for the dominance of the DNMT3a mutant clones amongst myeloid populations during infections (186). Human DNMT3a mutant hemopoietic stem cells exhibit similar defective interferon-γ induced differentiation. These studies demonstrate the manner in which interferon-γ signaling during chronic infection can drive DNMT3a loss of function in ARCH (186).

### 3.4. Immune effects on stem cells

Immune activity has long been known to suppress stem cell regeneration. In part this can be a useful activity because it helps to maintain stem cells in quiescence and thus protects their genomic material from replication-associated damage. When DNA breaks were introduced into immune cells in mice, changes of accelerated aging were noted in the immune system and also prominently in the liver, lung, and kidney (187). Alterations included increased oxidative levels and hydoxynonenal, oxidized glutathione, and oxidative DNA mutations (187). Thus, immune aging was shown to be causal for systemic aging. Immune activity works partly by inducing oxidative stress which includes the production of DNA breakages and also by induction of the SASP.

Aging and senescent skeletal muscle stem cell niches are known to become inflamed, and this suppresses satellite stem cell regenerative capacity (188). Similarly, obesity also triggers a systemic inflammatory state especially in the abdominal fat. In obese mice, PPARγ is downregulated and its support restores T_H_2 differentiation of CD4 T-cells and abrogates highly inflammatory T_H_17 T-cell differentiation which is seen in obese rather than lean mice (189). Interestingly in this study the PPARγ receptor was noted to heterodimerize with the RXRα receptor.

### 3.5. Transposable elements

Repeat elements comprise 54% of the human genome and transposable elements (TEs) make up 46% of the total genome length (190). TEs may be either class I retrotransposons, which encode a reverse transcriptase, or class II DNA transposons, which do not. Retrotransposons may be short interspersed repeat elements (SINEs), long interspersed repeat elements (LINEs), or long terminal repeat/endogenous retroviruses (LTR/ERV). Epigenetic remodeling in the cancer landscape can lead to mobilization of the TEs which are also ligands of innate immunity particularly of RNA via TLR3, RIG1, and MDA5 receptors, of DNA via TLR3/7/8, and cGAS (190). cGAS also binds DNA:RNA hybrids as may commonly occur during transcription (190). Low levels of DNA methylation derepress retroelements which then begin to jump through the genome and activate innate immune signaling whilst doing so (190). It has been shown that this process is particularly strongly activated in cancer genome atlas samples from stomach, bladder, liver, and head and neck squamous cancers. However, as mentioned, these mobile elements are immunoreactive and this immunogenicity can be re-purposed to therapeutic advantage for the development of cancer therapeutics (190). These TEs can also be used as a source of adjuvant antigens for CAR T-cell development for personalized cancer treatments. As cannabis is known to hypomethylate the genome and induce DNA breaks, these activities are highly relevant to cannabis medicine.

Class I transposable genomic elements are retrotransposons and include endogenous retroviruses (ERVs) and long interspersed repeat elements (LINE-1) which encode a reverse transcriptase and are thus auto-transcribing and mobilize across the genome through an RNA intermediate (191). One such LINE1 element in mice, known as Lx9c11, controls a Lx9c11-RegoS non-coding RNA which in turn controls hyperstimulated immune responses to viral infection and rescues infective lethality (191). In this case this transposable element is functional and suppresses immune reactivity.

### 3.6. Cancer immunogenomics

The immune system can either promote or suppress tumourigenesis (192). In either case it is clearly a major force impacting and sculpting tumor development, including selecting for those clones which are able to evade antitumor immunity (192). Chronic inflammation is a well-known precursor to tumor development in many tissue beds (10, 20, 193).

It was shown in pancreatic carcinogenesis that recurrent pancreatic inflammation raises IL-6 released from macrophages, which drives the expression of early growth factor 1 (EGF1) in acinar cells, which is the master transcription factor for neocarcinogenesis and widespread field change (194). The effect of these epigenetic rearrangements is to reduce zymogen secretion with subsequent bouts of pancreatitis and thus the degree of tissue damage of subsequent inflammatory bouts. Induction of oncogenic *Kras* expression similarly reduced the tissue injury caused by pancreatic inflammation, a finding which suggests that oncogenes might be actively selected due to their anti-inflammatory effect (194). Both EGF and *Kras* were identified in the epigenomic cannabis screen of Schrott et al. (86).

Other researchers compared gene expression from tumors in mice from two genetic backgrounds with and without a mature immune system. Surprisingly they found that the tumors from the mice in which the immune system was intact were enriched in tumor suppressor genes which was interpreted as indicating that suppression of these genes was important to assist with immune evasion (195). One pathway by which this occurred was CCL2 secretion which attracted immunosuppressive M2 macrophages. CCL2 was positively identified in the epigenomic cannabis screen of Schrott et al. (86).

Age-Related Clonal Hemopoiesis (ARCH) has been described above. It has also been shown that similar effects with clonal dominance happen in nearly all tissue beds (196). It is known to occur often through epigenomic changes that may silence one of the DNA oxidoreductase demethylases TET2/3 or DNMT3A (197). The rate of cancer development in ARCH is greatly elevated and this particularly relates to leukemias, which are 12-times elevated. Of these, acute myeloid leukemia (AML) is a lethal disease carrying a very high mortality rate. Disordered innate immune signaling is common in the development of leukemia and pre-leukemias. TRAF6 is a downstream adapter known for transducing signals from the TNF receptor. It was shown that TRAF6 co-mutation along with TET2 dysfunction caused a highly aggressive and transplantable AML-like disorder in mice (197). TRAF6 was found to function as an E3 ubiquitin ligase which attaches a ubiquitin molecule to K148 of Myc and thereby competes with its activating acetylation at this residue which induces constitutive Myc oncogene activity. TRAF6K148Ub does not affect Myc protein stability. This becomes important as Myc is known to interact with histone acetyltransferases and induce its own acetylation in numerous cancers (197). TRAF6 reduction has been identified in some human AML cases and these cases have a worse prognosis than others. In some cases this is due to increased methylation of the TRAF6 promoter (197). Leukemic blasts and TET2/3 deficient cells are proinflammatory and this may account for the higher incidence of atherosclerotic plaque and myocardial infarction observed in ARCH patients. Thus, ARCH is both fed by and feeds inflammation (196). The effects of inflammation on Myc expression may also be more general (196). Of relevance to cannabinoid medicine, TRAF6 was positively identified in the human sperm cannabis exposure epigenomic screen from Schrott et al. (86).

It is known that many infiltrating immunocytes into cancers are functionally downregulated and exhausted. The cause for this immune exhaustion is not well-understood and appears to be multifactorial in origin. A systematic screen of T-cells in mouse and human tumor models found that two remodeling complexes in the SWI/SNF ATP-dependent chromatin remodeling families INO80 and BAF were involved in exhaustion induction (198). The Arid1a complex in the BAF family was particularly implicated in maintaining the exhausted phenotype. Arid1a depletion increased the chromatin accessibility of active T-cell genes and improved anti-tumor immunity (198). Importantly, one of the activities of the INO80 complex is to resolve R-loops (DNA:RNA hybrids) which would otherwise stall DNA polymerase and thereby allow uninterrupted proliferation of cancer cells (199). When R-loops occur in the cytoplasm they are highly immunogenic via cGAS-STING (200). There were 210 DMRs identified in the epigenomic screen of Schrott for Arid1, many hits for members of the BAF complex, and five hits for the INO80 complex (86).

Tumor-infiltrating fibroblasts have also been shown to induce immune exhaustion (201). Irradiation is known to provoke an inflammatory response. In a murine model of rectal cancer, irradiation caused IL-1α release which both polarized fibroblasts toward an inflammatory phenotype and triggered DNA damage thereby predisposing them to P53-mediated senescence which in turn caused chemoradiotherapy resistance and disease progression (201). Blockade of IL-1α, prevention of fibroblast senescence, or senolytic therapy (ablation of senescent cells) rendered the tumors radiosensitive. The main antagonist of IL-1α is IL1RA (IL-1 receptor antagonist). Patients with a lower IL-1RA level had a worse prognosis (201). Thus, this pathway shows both the importance of immune-fibroblast interactions and defines an important therapeutic target for future work. There were eight DMRs identified in the Schrott epigenomic screen for cannabis exposure (86).

Similar findings were made in a mouse model of pancreatic cancer (202). Healthy fibroblasts could be transformed into immunosuppressive cancer-associated LRRC15^+^ myofibroblasts under the influence of TGFBR2 signaling (202). These cancer-associated fibroblasts (CAFs) suppressed the cytolytic activity of infiltrating CD8 T-cells. Deletion of these CAFs restored tumor sensitivity to checkpoint PD-L1 inhibition (202). There were 152 DMRs identified in the Schrott screen for TGFBRs (86).

It has also been shown that mouse haemopoietic stem cells (which are also lymphopoietic stem cells) present antigens to CD4 T-cells on class II MHC antigens as a means of quality surveillance. Cells that do not pass these checks are induced to differentiate and thereby eliminated from the stem cell pool (203, 204). Similar pathways operate in human haemopoietic stem cells. These findings demonstrate bidirectional signaling between HSCs and surveilling immunocytes and this process is also applied—and perturbed—in cancer (204).

### 3.7. Other immune actions

Microglia are known to play an important role in sculpting away unused dendrites and synapses from neurons (205–209) and also in controlling the growth of myelin sheaths laid down by oligodendroglial cells and their progenitors (210). Lung injury including smoking and lung infections are known to exacerbate autoimmune central nervous system disease (211). Using a rat model of experimental allergic encephalitis (EAE), researchers were able to show that prior tracheal insufflation of neomycin completely blocked the effects of subsequent intrathecal vaccination with myelin basic protein and the development of EAE (211). However, if the neomycin was not given EAE predictably developed. Neomycin shifted the pulmonary microbiota toward lipopolysaccharide releasing phyla (211). Changing the flora with polymyxin B removed the lipopolysaccharide releasing microflora and exacerbated EAE. These lung microbiota changes were associated with an alteration of brain macrophage from type I interferon releasing to type II interferon priming. These results demonstrated the existence of a lung-brain axis in the same way as a gut-brain axis has been demonstrated (211).

## 4. Synapses—Activity dependent synaptic plasticity

### 4.1. Cannabinoid psychological epidemiology

Documentation relating to an association between poor physical and mental health, somnolence, and unemployability of hashish devotees has long been a major issue in traditional societies and together constitute the reasons cannabis was out of favor to varying degrees in Islamic communities from the 9th to 18th centuries (212). Cannabis was first noted to be linked with insanity in western medical literature as long ago as 1930 (212). More recently, cannabis has been linked with adverse mental health outcomes in diverse psychiatric illnesses including an amotivational state (213–218), anxiety (219–225), depression (226–238), bipolar disorder (239–246), schizophrenia (235, 247–260), and suicidality (222, 230, 240, 243, 261–267). Whilst in previous decades the nature of these associations was somewhat controversial, these matters are now settled in the medical literature. Cannabis is also associated with a dependency and withdrawal syndrome which is more common in daily smokers, those who commence regular use in the early teenage years, and those who use cannabis products with higher THC concentrations where its incidence may rise to 50% of regular users (35, 218). Most recently, cannabis has been linked with mass homicide attacks, especially in the USA (230, 268–276). As has been astutely observed, “cannabis consumption and mental illness in adolescents and young adults are increasing in the United States” (277).

Pediatric autism spectrum disorder (ASD) has also been shown to be growing exponentially across the USA since 2000 (278–280). Increasing evidence supports a link with rising cannabis use, availability, and potency (281–289) and indeed in formal space-time and quantitative causal analyses cannabis has been shown to be the primary driver of this modern autism renaissance (290). Although cannabis or cannabidiol is frequently advocated as potential treatments for ASD, formal clinical trials have only produced mixed and conflicting results (291, 292).

Given that the fundamental units of computation in the brain are variously described as the neuron, the neuronal epigenome, subcellular organellar networks, the synapse, the dendrite, local cortical micronetworks, midbrain internuclear signaling, the machinery of the synaptic boutons, post-synaptic densities, and their associated astroglia, and since cannabinoid signaling disturbs all of these to varying degrees, it is appropriate to consider these issues in the present context to advance understanding of cannabinoid neurological synaptopathies.

### 4.2. Classical cannabinoid actions

The brain has 100 billion neurons and 10^15^ synapses a number which far surpasses the number of nodes in artificial intelligence networks (293, 294). However, this number can be multiplied by the many astrocytes which are the most numerous cells in the brain and also participate actively in tripartite synaptic trafficking in many ways (295, 296). Dendritic spines are believed to be a key site of much brain computation, learning, and memory, and are involved in essentially all brain functions and numerous neuropsychiatric disorders including depression, schizophrenia, and autism (293, 297, 298). Dendritic spines comprise 70% of the synapses in the cortex and occur with a frequency of 10 per micrometer (293).

Endocannabinoid (eCB)-dependent synaptic plasticity is highly dependent on synaptic activity and traffic. This is referred to as Hebbian learning (299). Synaptic plasticity can be induced by many stimulation protocols, especially those which involve repetitive short latency firing or that are associated with dopamine transients (293, 300, 301). Many neuronal subcompartments are known to be controlled by activity including the dendritic arbors, synaptic spines, axon initial segments, and presynaptic boutons (302). Endocannabinoid (eCB) induction of long-term potentiation has been identified in multiple brain regions including the hippocampus, striatum, amygdala, nucleus accumbens, nucleus of the solitary tract, ventral tegmental area, cerebellum, and the prefrontal, somatosensory, visual, and insular cortices, and spinal cord (303, 304). Synaptic potentiation is normally tightly controlled in both space and time and happens over a range of 0.5–1 micron and over seconds (293). eCBs are also involved in synaptic scaling and metaplasticity which scale the plasticity of the whole system generally (303).

Cannabinoids have long been known to mediate depolarization-induced suppression of inhibition (DSI) at inhibitory synapses (which is excitatory) (305, 306) and have also been shown to induce depolarization-induced excitation (DSE) at excitatory synapses (which is inhibitory) (307). Short- and long-term potentiation and depression of synaptic transmission have now been demonstrated (STE, STL, LTD, and LTP) (305, 308, 309). LTP mediated via CB1R is induced primarily post-synaptically and is demonstrated by increased receptor numbers and spine size, spine head area, and spine volume. It requires extended eCB stimulation (303). It can be induced by many stimulation protocols but is especially sensitive to induction by dopaminergic stimuli (300, 303). LTP is dependent on protein synthesis (300, 310). eCBs induce modifications of the active postsynaptic density zone matrix (303).

Cannabinoids are also known to play a key modulatory role on brain development during the neonatal and postnatal critical periods when GABAergic synapses are sculpting the excitatory and inhibitory brain circuits (307). Projections neurons from striasomes in the striatum form baskets around clusters of neurons in the ventral aspect of the Substantia nigra pars compacta; these structures are known as “striosome-dendron bouquets” (311). CB1Rs are necessary for the proper formation of these bouquets (311). Actin and spectrin form a regular repeating structure on axons (312, 313) which has been shown by super-resolution microscopy to form the framework into which CB1Rs are fitted (314–317). CB1Rs also play a critical role in the stabilization of nascent immature spines (318). All of these GABAergic changes are modulated by eCBs and act on somatostatin- and parvalbumin- positive GABAergic interneurons and also astrocytes (307). eCBs therefore play a critical role in the excitatory/inhibitory balance in both health and disease during development and maturity (307).

The activity of many receptor types drives eCB release and activation including mGluR1, mGluR5, M1/M3 muscarinic, 5HT_2_ serotoninergic, cholecystokinin (CCK_1_), orexin (OX_1_) and oxytocin (OT_1_), D_2_R dopaminergic, and α2-adrenergic receptors (319). Reward behavior has also been shown to control the strength of the hippocampus-nucleus accumbens synapses and the strength of these synapses is reduced by stress (320).

Human cannabis use disorder has been shown to reduce brain glucose oxygen consumption by PET scanning in cerebellum, orbitofrontal, and prefrontal cortices and basal ganglia (321). This has been linked with deficits in striatal dopamine release in cannabis dependence (322, 323). Downregulation of brain CB1Rs also occurred to a degree related to the years of cannabis smoking, which reversed after 4 weeks of monitored abstinence (324). This is the molecular representation of cannabinoid tolerance at the nanoscale. Thinning of cortical gray and white matter has also been demonstrated in cannabis dependence (325). Damage to white matter was shown innervating the posterior cingulate and parietal cortex, the basal ganglia, and the temporal cortex. Gray matter in the precuneus was thinned in a causal and dose-response manner (325). Regions with higher MAGL expression (the catabolic enzyme for 2AG) in human postmortem brains had more gray matter damage.

An fMRI study of cannabis use disorder showed increased connectivity between subcortical nuclei in the ventral striatum (housing the nucleus accumbens), the midbrain (accommodating the ventral tegmental area and substantia nigra), and lateral thalamus and brainstem (325). The level of suppression was noted to be related to the years of cannabis use and the degree of negative emotionality, depersonalization, and social alienation and perceived persecution experienced by patients (325). Thus, the heightened subcortical connectivity was believed to generate the negative emotionality which the reduced cortical connectivity was unable to ameliorate (325). It was also shown that cannabis use disorder patients had more recruitment of cerebral cortex to complete neurally demanding tasks together with less discrimination between cognitive and emotional processing (326). Disrupted thalamocortical connectivity in cannabis use disorder was documented in a further study from this group (327). Suppressed nucleus accumbens activity was seen in cannabis use disorder together with depressed corticostriatal and thalamocortical connectivity (327).

Significant structural effects on the brain from cannabis have been found in a meta-analysis of MRI studies and include relative atrophy of the hippocampus and medial and lateral orbitofrontal cortices, which have been found with effect sizes measured as standardized mean differences of 0.14, 0.30, and 0.19, respectively (328).

The clear convergence in this data from the world leading group at Brookhaven National Laboratory's state of the art scanners between negative emotionality, psychological persecutory complexes, social alienation and estrangement, impaired cortical control of subcortical hedonic drives, and confusion between emotional and cognitive processes and difficulty performing neural computation tasks and the above described predisposition to self-directed and other-directed acts of violence is both noteworthy and of serious concern. Data strongly suggest a causal pathway to irrational, ill-considered, and confused psychological states and behaviors.

### 4.3. Non-classical endocannabinoid actions

Whilst eCB release is usually considered to be phasic and eCBs are described as being synthesized on demand, administration of eCB antagonists and genetic studies clearly demonstrates that there is also tonic eCB tone (305, 319). As well as classically described coupling of CB1Rs and CB2Rs to G_i_ proteins, non-classical coupling with G_S_ and G_0_ G-proteins and β-arrestins is also documented (305). CB2Rs have also been identified on endoplasmic reticulum (305). When a newer and more sensitive marker for labeling astrocytes was employed, it was shown that 12% of the cerebral CB1R staining occurred on astrocytes (329). Astrocytes also display CB1Rs and eCB release onto astrocytes causes increased release of the gliotransmitters glutamate, D-serine, and adenosine, causing increased LTP (296, 305, 330).

### 4.4. Spine types

Synaptic spines can be of three types: filipodia, small, and large (293, 302). There is a tight relationship between spine structure and function with LTP increasing and LTD decreasing spine size (302). At the nanoscale, spines fluctuate in size over minutes, hours, and days, with the largest spines being the most stable (331). Filipodia are small thin spines which are usually transient with only 3% lasting for 1 day (293, 297). Spines are rapidly responsive to stimulation within 1 min (293). If they are contacted by an axon, they can become a small spine. Increasing evidence implicates pathology at spines in many diseases including anxiety, depression, autism, intellectual disability, schizophrenia, and bipolar disorder (302). LTP leads to an increase in the size, number, and stability of spines, actin polymerization, an increase in the number of AMPARs and their surface trafficking, and the size of the post-synaptic density matrix (302). NMDA glutamate receptors allow ingress of extracellular calcium and via calcicalmodulin kinase II (CAMK2) are key to the orchestration of the changes of synaptic growth and plasticity (293, 302). Compound synapses also occur of two to six synapses and are more stable than single synapses (293). Large synapses are more stable as they are further from the pruning boundary (293).

Structural studies show an increase in the number and density of spines in the frontal, temporal, and parietal lobes in Layer II in autism and a reduction in schizophrenia (302). GWAS studies of neuropsychiatric disorders most prominently show a reduction in postsynaptic glutamatergic signaling and also changes in cytoskeletal organization, chromatin modifiers, and transcription regulation (302).

Also key to the organization of the postsynpatic bouton are the scaffolding proteins including shank and homer; actin which forms the basic structural protein of the spine, including the “knob” and the neck of the bouton, arrests passing ribosomes toward the bouton, and directs microtubules into it to carry their mitochondria as cargo to supply energy (331); and PSD95/DLG4, which forms the plate-like area subjacent to the synaptic cleft into which AMPARs and NMDARs are inserted and bound (302, 331). Cofilin (CFL1 gene) is a key molecule with severs actin and induces the formation of branches and new actin polymers (331). PICK1 and oligophrenin (OPHN1 gene) are similarly key anchoring and actin controlling proteins (331). Calcium channels such as CACNA1C are clearly key since many of the changes described are calcium-induced, as are the small Ras GTPase family including Ras and RAC1 which induce spine formation, enlargement, maturation, and stabilization, and RhoA and RAP which have the opposite effect (302). RhoA and RAC1 also control the actin cytoskeleton. The Ras family is also a well-known oncogene (302). Neurexin and neuroligin are key transsynaptic scaffolding proteins (331). Shank proteins are highly implicated in autism spectrum disorder (ASD), which highlights ASD in part as a synaptopathy (332). Genes active in neurogenesis, chromatin modification, and synaptic function have also been implicated in autism (333).

It is therefore of interest to observe that many of these key receptors were positively identified in the epigenomic cannabis screen of Schrott et al. (86). including AMPARs (GRIA 132 DMR hits), NMDARs (GRIN 26 hits), metabotropic glutamate receptors (GRM 122 hits), GABA receptors (GABR 143 hits), dopamine receptors (DRD 17 hits), orexin/hypocretin receptors (HCRT 1 hit), μ-opioid (ORPM, 5 hits), and δ-opioid (ORPD, 5 hits). Of the scaffolding and framework molecules, the following were identified: actin (207 hits), tubulin (106 hits), cofilin (CFL1, one hit), PICK1 (one hit), oligophrenin (one hit), DLG/PSD95 (37 hits), SHANK (6 hits), HOMER (2 hits), calcium channel CACNA1 (46 hits), CAMK2 (5 hits), Ras (146 hits), RhoA (1 hit), RAP (438 hits), neurexin (27 hits), and neuroligin (10 hits) (86). When genes involved in depression were intersected with genes modified by cannabis, multiple genes were found in common in mouse, rat, and human studies (334). The autism candidate gene DLGAP2 was found to be altered in rat and human sperm after cannabis exposure (282).

### 4.5. Synaptoenergetics

Activities at the presynaptic terminal are generally heavily energy dependent. Terminal depolarization by an action potential causes calcium to flow into the terminal and the exocytosis of neurotransmitters from presynaptic vesicles (294, 335). Restoration of ionic gradients, synaptic cargo transport, synapse assembly and maintenance, pumping out presynaptic calcium, and synaptic vesicle refiling and recycling all require ATP. Indeed, it has been estimated that the recycling of a single glutamate synaptic vesicle requires 20,000 ATP molecules and that maintenance of the terminal at a steady state requires the presence of 1,000,000 ATP molecules (294).

Whilst the human brain is only 2% of body weight, it has been estimated to consume 20% of the energy budget. Glucose may be a major source of brain energy and 55% of the brain's glucose is consumed at axon terminals (294). Running the Na^+^/K^+^ and Ca^2+^ pumps which maintain the ionic gradients is a major energy requirement.

ATP is generated at the nanoscale in cellular microdomains. On demand glycolytic energy can be released from glycolytic enzymes mounted on the plasmalemma, within axonal transport cargos, and on synaptic vesicles to supply motor ATPases and ion transport ATPases with fast on-board refueling. Energy is also supplied from astrocytes by the lactate shuttle. Both mitochondrial oxidative phosphorylation and glycolysis can be upregulated in periods of intense activity (294).

A large calcium influx occurs into the presynaptic terminal with depolarization. This is taken up partly by the mitochondrial voltage-dependent anion selective channels and calcium uniporter of the inner and outer membranes and this has the effect of powerfully upregulating mitochondrial metabolism by stimulating its many calcium sensitive enzymes (294). Calcium also increases the surface area of cristae and cytochrome oxidase activity and induces a proteomic adjustment in favor of increased energy production. The anti-apoptotic protein BAD also stimulates mitochondrial biomass, enhances energy production, and reduces the leak from the F_1_F_0_-ATPase, thus enhancing the efficiency of energy production (294).

Mitochondrial biogenesis is a key mechanism by which mitochondria undergo fission and fusion in order to increase energy delivery to the presynapse. Mitochondria are trafficked along dendrites and axons and are typically carried by a kinesin at one end and a dynein at the other, which move the mitochondria toward the plus and minus ends of the microtubule, respectively (294). Mitochondria trafficking along microtubules are arrested by local high calcium levels. Short-range movements of mitochondria within the spine are conducted by myosin motors (294).

Neuronal synaptic terminals possess a master energy sensor called AMP kinase (AMPK) which drives many of the changes to increase energy production for periods of high energy requirements (294). AMPK has multiple presynaptic activities including adapting glycolysis and mitochondrial respiration, sustaining LTP and high frequency stimulation, facilitating mitochondrial transport and presynaptic distribution, and driving the accumulation of mitochondria within the axonal compartment. Importantly, this is the same key molecule that is well-known to sense energy and is a key controller of lifespan in lower organisms (72, 336–348).

Thus, mitochondria play a dual role in the presynaptic terminal by virtue of their interrelated roles for energy supply and calcium buffering. Failure of synaptoenergetics has been shown to play a key role in diseases such as Alzheimer's and Parkinson's disease and in motor neuron disease (294).

Two of the key mitochondrial fusion proteins, OPA1 and MFN1, were identified in the cannabis epigenomic screen of Schrott with three and one DMR identified, respectively (86). There were 217 DMRs in the Schrott screen for kinesins, 16 for dyneins, and four for dynactin, the regulatory partner of dyneins (86). AMPK was identified in the Schrott screen with five DMR hits (86).

Endoplasmic reticulum (ER) also play an important role in presynaptic calcium control as they are able to actively acquire calcium from the cytoplasm and hold the largest cellular stores of this cation (349). Moreover, ER are electrically excitable and can be triggered by action potentials (349). ER are also a major site of protein synthesis as they house ribosomes. Lysosomes also concentrate calcium, and the release of the calcium from just one lysosome has been shown to elevate cytosolic calcium by two orders of magnitude (349).

### 4.6. Brain-derived neurotrophic factor—Extracellular vesicles

Extracellular vesicles (EVs) are known to originate in intracellular endosomes.

Brain EVs promote communication between neurons and non-neuronal cells, prune excitatory synapses, and modulate inhibitory synapses the transmission of viruses, and prion protein aggregates in preclinical Alzheimer's disease models (350, 351).

They are also useful for critical events such as during rapid maturation of the neuronal proteome such as in dendrite maturation and growth cone steering (350).

BDNF modulated both eCB LTD and LTP in the hippocampus, neocortex, ventral tegmental area, and striatum via its high affinity TrkB receptor. BDNF stimulates 2-AG release and CB1R activation and DSI causing increased glutamatergic LTP in the neocortex (304). BDNF release leads to the release of neuronal vesicles from the post-synaptic terminal carrying miRNAs (miR-132-5p, miR-218-5p, and miR-690) which induce clustering of the synaptic vesicle in the presynaptic terminal, increased calcium transients in the post-synaptic terminal, regulate the abundance of synaptogenic transcripts, increase BDNF-dependent dendrite outgrowth, increase synapse density, and increase neuronal network bursting and synchronized activity and connectivity (350).

BDNF was necessary and sufficient for the formation of excitatory synapse formation in the hippocampus. EVs induce synchronous neuronal network firing (350).

Of relevance to cannabinoid medicine, THC has been shown to epigenomically downregulate BDNF mRNA and protein expression in the ventral dentate gyrus (352). BDNF was identified by three DMRs in the Schrott cannabis epigenomic database and the high affinity BDNF receptor TrkB (gene NRTK2) was identified by 33 DMRs in this database (86).

### 4.7. Epigenetic mechanisms

From the above discussion, it is clear that whilst topographically the synapse is considered the unit of computation, changes that are localized to the synapse must clearly be coordinated with events in the cell. As mentioned, the apparatus of the synapse involves over 500 proteins which must be synthesized locally or imported and there is a high demand for local energy. Therefore, ribosomes and mitochondria are re-routed as they travel along dendritic microtubules and imported into larger spines (293). It has been convincingly demonstrated that memory formation (involving spine enlargement, consolidation, and maturation) requires protein synthesis and frequently occurs overnight (293, 310). Spine enlargement and maturation involves several signaling cascades including changed distribution of glutamate receptors and synaptic adhesion proteins (18).

From these observations, it becomes clear that epigenomic controls of protein expression must be coordinated with events at the synapse for the whole system to work cohesively. That is, memories which are encoded epigenomically must be coordinated and synergize with local synaptically encoded memories (18). Thus, disorders of DNA methylation, histone methylation, histone acetylation, CBP, CREB1, and HDAC5 have all been linked with impairments of learning and memory (18).

Signaling from the synapse to the nucleus can occur via activation-induced CREB-regulated transcriptional coactivator 1 (CRTC1). Strong training also induces the brain-specific FGF1B, which is required for CA3-CA1 learning in the hippocampus (18). FGF1B transcription in the nucleus is induced by CRTC1, which displaces the transcriptional co-repressor HDAC3 nuclear receptor corepressor (NCOR) complex, leaving phosphorylated CREB-CBP to bind in its place, thereby activating FGF1B transcription (18). Strong training-induced continued CRTC1 expression displaced CBP and drove the insertion of the HAT KAT5, which induces sustained FGF1B expression. KAT5 substitution was required for hippocampal synaptic plasticity and memory enhancement (18). Afadin is another protein that shuttles from the synapse to the nucleus and induces epigenetic change, which in this case is H2S10 which opens heterochromatin to allow a more permissive chromatin state for gene transcription (18). From remarks made above, it is also likely that extrasynaptic vesicles also traffic information into the dendrite and neuronal soma.

It is noted that, in the Schrott cannabis epigenomic screen, there were two hits for CRTC1 and six hits for NCOR (86).

Signaling from the nucleus to the synapse occurs by virtue of control of AMPAR synthesis during critical periods, stress, and drug exposure. Stress-induced cortisol release which increased HDAC2 occupancy of the G9a promoter and impacts on E3 ubiquitin ligase leads to increased ubiquitinoylation of AMPARs and their down regulation. Similarly, an epigenetic cascade of histone acetylation and H3K9 trimethylation can control the alternate splicing of neurexin-1 which controls its affinity for post-synaptic binding partners and thus synaptic activity and remodeling (18).

In this way detailed, complex, and profound coordination between the epigenomic machinery of the nucleus and the structural machinery of the synapse is orchestrated.

One elegant example of these changes is with PRKCZ. PRKCZ has been well-characterized as being involved in long-term potentiation and synaptic strengthening (353). PRKCZ abnormalities have been implicated in alcoholism, depression, and Alzheimer's disease. DNA methylation of the internal intronic promoter carrying a CREB -binding site of the human brain-specific PRKCZ gene was noted to be controlled by DNA methylation (353). Methylation of this site caused reduced CREB binding and downregulation of PRKCZ transcription (353). CREB has been implicated in both short- and long-term synaptic potentiation and adult neurogenesis and controls cassettes of genes that are involved in these processes (353). DNA methylation prevents CREB docking at the site (353). Similar control mechanisms were found in three other genes involved in long-term potentiation and neuronal differentiation from stem cells (LRRTM2, NEUROD2, and FAM163B) (353). PRKCZ recruits AMPARs to the post-synaptic density and LRRTM2 stabilizes them there.

PRKCZ was identified by two DMRs in the Schrott cannabis epigenomic screen (86).

### 4.8. Clinical implications

It is thus apparent that cannabinoids can modulate brain synaptic signaling at many levels including neuronal, neuronal mitochondria, neuronal and astroglial epigenomic, subcellular trafficking, and pre- and post- synaptic levels. and modulate neurotransmission in several subtle and classical directions including impacting both long-term synaptic depression and potentiation. Therefore, these various and interrelated mechanisms form the neurobiological substrate for the now documented widely diverse clinical syndromic phenomenology of adult and inheritable pediatric neurotoxicity described in the opening paragraphs.

With this widespread disruption of neural activity at many levels, it becomes clear how cannabis exposure might be related to so many diverse neuropsychiatric syndromes including depression (227, 229, 230), anxiety (224, 225, 230), schizophrenia (354–358), bipolar affective disorder (239, 240, 245), and autistic spectrum disorder (278, 279, 290, 359).

The putatively causal pathway from confused thinking, heightened subcortical hedonic drive, impaired cortical control of subcortical activity, and underlying highly negative emotional state with advanced social alienation and persecutory perseverative thinking is of particular concern in terms of contemporary issues with cannabis-related violence, suicides, and homicides. In that this pathway has been well-described in reports from the Brookhaven National Laboratories an internationally pathfinding group in the brain scanning of neuropsychiatric disorders (323, 325–328, 360–363), this evidence can only be considered state of the art.

It is also of great interest that AMPK, which is one of the best established molecules with an important role in aging medicine (364–366), is also a key and central regulator of synaptoenergetics at the presynapse (294).

A real concern has been expressed that overwhelming this delicate synaptic and epigenomic machinery of DNA methylation, histone modifications, and microRNAs with exogenous phytocannabinoids will disrupt the delicate spatiotemporally defined patterns of eCB-controlled systems which maintain normal brain function and development (367). Given that mitochondria play such a key role in synaptoenergetics, it is clear that the general disruption of mitochondrial metabolism will necessarily perturb both neuronal and synaptic activity and epigenomic regulation. Given that synaptic dysregulation is a key component of most neuropsychiatric disorders, these disruptions are likely to have far reaching and protean manifestations (293, 297, 298). In view of the fact that both the brain and mitochondria also control systemic aging and disease (364, 365) these impacts are likely to ramify beyond even psychiatry to the general domain of whole-body health.

## 5. Conclusion

This wide-ranging overview considered in Parts 1 and 2 of this review has considered the manner in which interrelated, interlocking, and interdependent deleterious changes have been shown to be induced by cannabinoids in multiple mechanistic layers including the genome, epigenome, metabolome, and immunome. Changes in synaptic plasticity illustrate and highlight these metabolic, proteomic, and epigenomic alterations. It is emphasized that these changes do not occur in isolation but in a coordinated cross-platform manner. A striking feature of the data is its cross-disciplinary concordance so that, for example, CB1R downregulation occurring in cannabis dependence and first demonstrated by autoradiography in brain slices has now been confirmed by super-resolution microscopy and has been shown to be reflected in blunted hedonic drive in the midbrain and ventral tegmental area and in inhibited cortical connectivity from the frontal lobes. Synaptic alterations are necessarily supported by metabolic and epigenomic alterations and synergize with altered dopaminergic drive, all of which impact on learning, emotionality, behavior, and memory. The evidence for altered and confused behavior including persecutory and emotively driven thinking from the Brookhaven scans is relevant to modern considerations of cannabis-associated self- and other- directed violence.

There is an impressive synergy between the altered immunome, metabolome, and epigenome that fits with the altered gene expression seen in clinical syndromes such as diverse cancers and numerous congenital anomalies identified in modern large-scale epidemiological studies (2–20, 69, 368–385) and the genomic, epigenomic, metabolomic, and immunomic changes of aging (386–391) which together reflect altered and disrupted epigenomic-metabolomic regulation. Clearly, the well-established inhibition of mitochondrial oxidation is compounded by the immunostimulatory CB1R-mediated cannabinoid actions and together they have greatly amplified downstream effects.

Thus, it is important to hold a proper appreciation of the epigenomically mediated alterations of three-dimensional genomic architecture, gene expression, and epitranscriptome in mind in order to foster a comprehensive appreciation of multifaceted and diverse cannabinoid pathophysiology. Moreover, given the rapid growth of the cannabis industry worldwide, there is a pressing and urgent need to complete single cell three dimensional chromosomal conformation capture (low input Hi-C methods) and associated mass spectrometry histone modification, DNA methylation, whole genome, whole epigenome, enhancer, superenhancer, superanchor, and m^6^A epitranscriptome studies in many brain areas, and in the heart, liver, respiratory tract, immune, muscle, testicular, and ovarian tissues in a timely manner.

## Author contributions

AR conceived the idea, performed the literature review, and wrote the first draft. GH added meaningful intellectual input, edited the first draft, provided project supervision and support, curated resources, and supervised the conduct of the project. All authors contributed to the article and approved the submitted version.

## Glossary

NF-κB, Nuclear Factor kappaB, the key master transcription factor of the immune system; ROS, Reactive Oxygen Species; also known as oxygen radical; includes hydrogen peroxide, superoxide anion, and the hydroxyl radical; HIF1α, Hypoxia Inducible Factor 1; FGF21, Fibroblast Growth Factor 21; GDF15, Growth Differentiation Factor 15; SIRT1, Silencer of Information Transfer 1; AMPK, Adenosine Monophosphate Kinase; TFS1, Stress-activated transcription factor ATFS-1; ATFS-1, Activating Transcription Factor Associated With Stress-1; GPR, G-Protein Receptor; HDAC, Histone Deacetylase; HMGB1, High Molecular Weight Group Box protein 1; ARG1, Arginase 1—catalyzes the hydrolysis of arginine to ornithine and urea; CCL5, C-C Motif Chemokine Ligand 5; SLC16A, Solute Carrier Family 16, Member 1 (Monocarboxylic Acid Transporter 1); NFAT, Nuclear Factor of Activated T Cells; VEGF, Vascular Endothelial Growth Factor; Kla, K is the abbreviation for lysine and la stands for lactate; ACAT1, Acetyl-CoA Aceteyltransferase 1; CXCL9, C-X-C Motif Chemokine Ligand 9; cGAS-STING, cyclic AMP—cyclic GMP—Stimulator of INterferon Gamma (STING); R-loops are DNA, RNA hybrids and can arise during DNA transcription; Stat, Signal Transducer and Activator of Transcription; RelB, RELB Proto-Oncogene, NF-KB Subunit; NF-κB, Nuclear Factor kappa B; JNK, c-Jun—N-terminal kinase; ASK, Apoptosis Signal Regulating Kinase 1/Mitogen-Activated Protein Kinase Kinase Kinase 5/MAPKKK5/MAP3K5; ABA, Gamma Amino-Butyric Acid; DAMP, Damage-Associated Molecular Pattern; PAMPm, Pattern-Associated Molecular Pattern; ARCH, Age Related Clonal Haematopoiesis; SASP, Senescence-Associated Secretory Phenotype; LR, Toll-Like Receptor; RIG1, Retinoic Acid Inducible Gene 1; MDA5, Melanoma Differentiation-Associated Protein 5; cGAS, cyclic GMP – cyclic AMP Synthase; CAR T-cells, Chimeric Antigen Receptor T-cell lymphocytes; CCL2, C-C Motif Chemokine ligand/MCP-1, Macrophage Chemotactic Protein 1; TRAF6, TNF Receptor-Associated Factor 6; SWI/SNF, SWItch/Sucrose Non-Fermentable chromatin remodeling complexes; INO80, INO80 Complex ATPase Subunit; BAF complex, the mammalian equivalent of SWI/SNF in yeast; Includes BANF1, BAF Nuclear Assembly Factor 1; ARID1a, AT-Rich Interaction Domain 1A; LRRC15, Leucine-Rich Repeat Containing 15; TGFBR2, Transforming Growth Factor Beta Receptor 2; mGlu1, Glutamate Metabotropic Receptor 1/GRM1; MAGL, MonoAcyl Glycerol Lipase the enzyme which metabolizes 2-arachidonyl glycerol; AMPAR, α-Amino-3-hydroxy-5-Methyl-4-isoxazole Propionic Acid glutamate Receptor; PSD95, Post-Synaptic Density 95; DLG4, Discs Large MAGUK Scaffold Protein 4; PICK1, Protein Interacting With PRKCA 1; CACNA1C, Calcium Voltage-Gated Channel Subunit Alpha1 C; Ras, KRAS Proto-Oncogene, GTPase; RAC1, Rac Family Small GTPase 1; RhoA, Ras Homolog Family Member A; RAP - RAP, Member of RAS Oncogene Family; AMPK, AMP kinase/PRKAA1, Protein Kinase AMP-Activated Catalytic Subunit Alpha 1; OPA1, OPA1 Mitochondrial Dynamin Like GTPase / Optic Atrophy 1 (Autosomal Dominant); MFN1, Mitofusin 1; BDNF, Brain-Derived Neurotrophic Factor; CBP, CREB Binding Protein; HDAC5, Histone DeACetylase 5; FGF1B, Fibroblast Growth Factor 1B; PRKCZ, Protein Kinase C Zeta; CREB1, cAMP Response Element Binding Protein 1; LRRTM2, Leucine Rich Repeat Transmembrane Neuronal 2; NEUROD2, Neuronal Differentiation 2; FAM163B, Family With Sequence Similarity 163 Member B.
